# Artificial intelligence in breast cancer screening: A systematic review and meta-analysis of integration strategies^☆^

**DOI:** 10.1016/j.ejro.2026.100727

**Published:** 2026-01-10

**Authors:** Eloïse Sossavi, Catherine Roy, Sébastien Molière

**Affiliations:** Radiology Department, Hautepierre Hospital, Strasbourg University Hospital, Strasbourg, France

**Keywords:** Breast cancer, Screening, Artificial Intelligence

## Abstract

**Objective:**

To compare AI-augmented and conventional double reading in organised breast-cancer screening with respect to cancer-detection rate (CDR), recall rate, and radiologist workload.

**Methods:**

We conducted a systematic review and random-effects meta-analysis of 13 prospective and retrospective studies (1.03 million screens) from 2017 to 2024 that embedded commercial or research AI into population-based digital mammography or tomosynthesis programmes. Eligible studies included ≥ 10,000 screens (or ≥100 cancers) and reported CDR, recalls, and/or workload metrics. We extracted cancer and recall counts and calculated risk ratios (RRs) for AI-augmented versus double reading, overall and by integration model: independent second reader, gate-keeper/decision-referral triage, and concurrent overlay.

**Results:**

Overall, AI-augmented protocols achieved CDR parity (RR 1.01; 95 % CI 0.96–1.07) and no significant change in recalls (RR 1.00; 95 % CI 0.88–1.15). Triage models preserved CDR (RR 1.02; 95 % CI 0.98–1.07) while reducing recalls by 11 % (RR 0.89; 95 % CI 0.82–0.96) and cutting initial reads by 44–70 %. Independent-reader workflows maintained CDR (RR 0.98; 95 % CI 0.92–1.05) but showed variable recall effects (RR 1.12; 95 % CI 0.90–1.39) driven by arbitration logic and threshold choices. Concurrent overlay (two studies) indicated possible sensitivity gains (RR 1.31; 95 % CI 0.90–1.91) without higher recall rates, though precision was limited.

**Conclusions:**

AI integration can match conventional double reading in detection performance, but its impact on workflow depends on the chosen model. Triage-based approaches consistently lower radiologist workload and recalls without compromising sensitivity, whereas replacing a second reader may simply shift effort to arbitration. Future implementation should focus on workflow-aware metrics and prospective threshold validation.

## 1 Introduction

Screening mammography is proven to reduce breast-cancer mortality [1], yet even in mature programmes its performance is constrained by human perception error and the logistical burden of reading millions of examinations. Double reading—standard in many European systems—improves sensitivity but doubles radiologist workload [2], a growing problem as experienced readers become scarce. Recent EUSOBI (European Society Of Breast Imaging) - endorsed guidelines call for a risk-stratified approach using supplemental modalities and new technologies to optimize benefit-to-harm and cost-effectiveness [3].

Deep-learning–based artificial-intelligence (AI) algorithms are now commercially available and claimed to (i) match or exceed radiologist accuracy and (ii) cut the number of human reads. A recent meta-analysis [4] pooled > 1 million examinations and showed that stand-alone AI can equal a single radiologist’s AUC (Area Under the Curve), but that conclusion was derived mainly from enriched reader studies or retrospective single-reader cohorts.

A central implementation question for screening services is how AI performs when embedded within established double-reading pathways, and how each integration strategy affects recall, arbitration, and workload. Early implementation studies suggest three distinct integration models: (i) independent second reader, (ii) triage, (iii) concurrent overlay **(**AI marks are shown to both radiologists during their routine reads, acting as real-time decision support). These configurations raise distinct questions about diagnostic gain, recall burden and workforce relief—issues that remain unsettled both in the literature and in public opinion, where acceptance of screening AI is contingent on oversight, equity and demonstrable benefit [5], [6], [7].

We aimed to determine how AI integration within population breast-cancer screening compares with conventional double reading in terms of cancer-detection, recall, and workload. The present systematic review therefore restricts inclusion to studies—prospective or rigorously simulated—that embed AI inside a breast cancer screening population. We pooled cancer-detection rate, recall rate, and workload outcomes across three predefined integration models to identify where AI provides measurable clinical benefit and where trade-offs remain.

## 2 Methods

This systematic review was designed, conducted, and reported in accordance with the Preferred Reporting Items for Systematic Reviews and Meta-Analyses (PRISMA) 2020 guidelines [8] and the Quality Assessment of Diagnostic Accuracy Studies–Artificial Intelligence (QUADAS-AI) tool [9].

### 2.1 Search strategy

A systematic literature search was conducted using the PubMed database, covering original articles published from January 2017 to January 2024. The search terms used were the following: « artificial intelligence » AND « breast » AND « screening » AND (« mammography » OR « tomosynthesis » OR « DBT » OR « digital breast tomosynthesis ») NOT « review » .

### 2.2 Eligibility criteria

#### 2.2.1 Design

Prospective service studies, randomised or non-randomised, and retrospective cohort or simulation studies.

#### 2.2.2 Population

Consecutive women attending organised breast-cancer screening with digital mammography (DM) or digital breast tomosynthesis (DBT).

#### 2.2.3 Intervention

Commercial or research AI providing an independent or supportive read inside the screening workflow.

#### 2.2.4 Comparator

Conventional single-reader or double-reader screening programme.

#### 2.2.5 Size filter

Retrospective and simulation studies had to include ≥ 10,000 screening examinations or ≥ 100 screen-detected cancers. The 10,000 screening size limit was required for several reasons. First, at the typical cancer prevalence of 0.5 % in biennial European programmes, 10,000 screens yield approximately 50 cancers, thus providing a 95 % binomial confidence interval of ± 0.14 %age points around the cancer-detection rate, which is adequate precision for programme-level benchmarking. Second, although regulatory bodies do not define a specific numeric lower bound, they emphasise that validation datasets must include a “sufficient number of cases such that confidence intervals can be characterized”. [10] Finally, publicly-available mammography-screening databases developed for AI-research workflows are frequently in the neighbourhood of ten thousand or more screening examinations, for example, the DDSM dataset and the RSNA Screening Mammography Breast Cancer Detection AI Challenge [11], [12]. Prospective randomized control trials (RCTs) were included regardless of size.

#### 2.2.6 Outcomes

Any of: cancer detection rate (CDR), recall, Sensitivity, Specificity, Positive Predictive Value, Negative Predictive Value, Area Under the Curve (AUC), recall rate (RR), number of reads averted, reading time.

#### 2.2.7 Exclusion

(1) Enriched data sets with > 10 % cancer prevalence unless results re-weighted to population prevalence; (2) studies using AI only for segmentation or preprocessing.

### 2.3 Study selection

One reviewer (E.S.) screened titles/abstracts; full texts that met criteria were assessed independently by two radiologists (E.S., S.M.). Disagreements were resolved by consensus.

### 2.4 Data extraction

A piloted form captured:

• Bibliographic details (authors, year, country).
• Design (prospective/retrospective, RCT, simulation).
• Population (number of screens & women, age, cancer prevalence).
• Screening modality (DM, DBT, or both).
• AI system (name, version, operating threshold, integration method).
• Radiologist characteristics (number, experience).
• Performance metrics: AUC, sensitivity, specificity, CDR, recall rate, positive predictive value, negative predictive value
• Workload indicators (reads averted, reading time, arbitration volume).
• Sub-group results (e.g., breast density).
• Risk-of-bias signalling (QUADAS-AI) as assessed by E.S. and S.M. in consensus.

### 2.5 Quantitative synthesis

#### 2.5.1 Effect measure

Risk ratio (RR) of AI-augmented versus conventional double reading for CDR (cancers per 1 000 screens) and recall rate (% screens recalled). When studies provided raw numerators and denominators (e.g., number of screen-detected cancers and total screens, or number of abnormal examinations), we calculated cancer-detection rate (per 1 000 screens) and recall rate (percentage) ourselves.

#### 2.5.2 Meta-analysis

We pooled study-specific log-risk ratios using a DerSimonian–Laird random-effects model and quantified between-study heterogeneity with the I² statistic. Because several integration-model subgroups contained few studies, we performed a sensitivity analysis using a Hartung–Knapp adjustment to the random-effects model.

Prespecified subgroups: Pooled estimates were additionally generated for each AI-integration category:

− Independent second reader – AI replaces one of two human votes.
− Gate-keeper / supporting reader – AI triages cases that would otherwise receive a second read.
− Concurrent overlay – AI marks displayed to each radiologist in a single-reader programme (typical in the U.S.) or during the first read of a two-reader programme.

Several studies evaluated multiple AI-integration scenarios (e.g., different triage thresholds, independent-reader configurations, or concurrent-overlay variants). To avoid double-counting the same underlying population, we pre-specified one arm per study based on clinical relevance and consistency with real-world deployment: (i) in triage studies we selected the primary gate-keeper or allocation workflow; (ii) in independent-reader designs we retained the R1 +AI arbitration arm designated by the authors as their intended-use configuration; (iii) in threshold-based studies we chose the threshold recommended for clinical practice. All other arms from the same underlying screening cohort were excluded from pooling.

Analyses were performed in Python 3.11 using *pandas*, *numpy* and *matplotlib*.

## 3 Results

### 3.1 Study selection and general characteristics

**Fig. 1:**
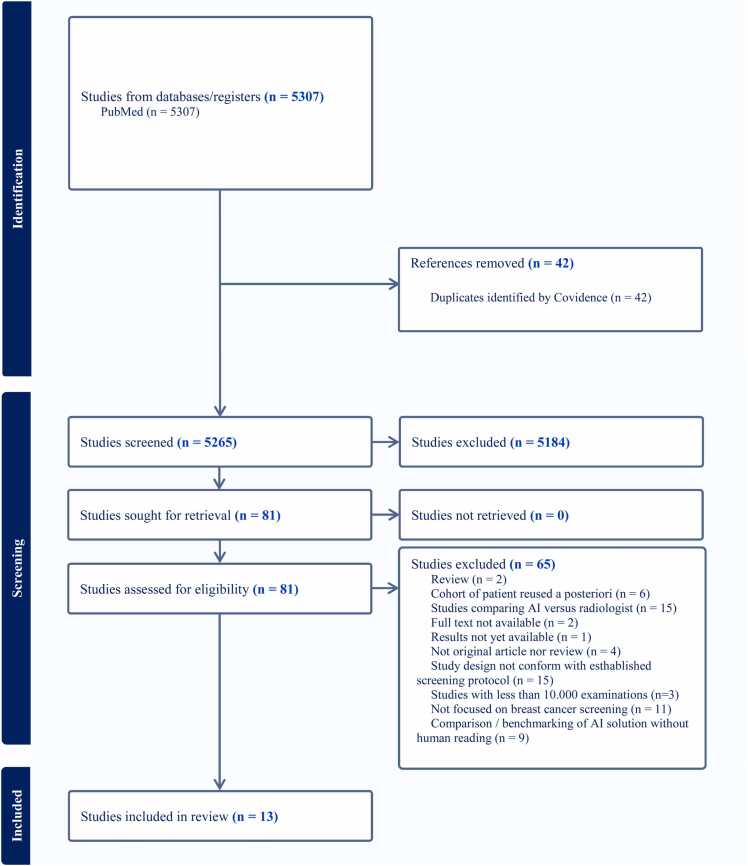
PRISMA 2020 flow diagram of the study selection process. The search identified 5307 unique records, of which 81 were assessed for eligibility, resulting in 13 studies included in the final meta-analysis. PRISMA stands for Preferred Reporting Items for Systematic Reviews and Meta-Analyses.

The search yielded 5307 unique records after duplicate removal (Fig. 1). Title- and abstract-screening excluded 5184 papers; 81 full texts were assessed and 13 studies met every inclusion criterion (PRISMA flow-diagram, Fig. 1). Two studies were excluded for sample size < 10 000 screens [13], [14].

The thirteen studies encompass approximately one million screening examinations from double-reading breast screening programmes in Europe (n = 10) [15], [16], [17], [18], [19], [20], [21], [22], [23], [24] and Australia (n = 1) [25] and from single-reader U.S. screening programmes (n = 2) [26], [27]. Two were prospective service evaluations or randomised trials [16], [19]; the remainder were simulated retrospective cohorts. Ten studies analysed 2-D digital mammography only [16], [18], [19], [20], [21], [22], [23], [24], [25], [27] and three studies evaluated AI on digital breast tomosynthesis [15], [17], [26] - one study applied AI concurrently to both digital mammography and tomosynthesis [17].

### 3.2 AI integration within double reading

Across the thirteen publications we reviewed, artificial intelligence was embedded in the screening pathway in three broad ways—(i) AI as a second, independent reader, (ii) AI-led triage that decides how many humans, if any, will read the examination, and (iii) AI that merely annotates the image while humans retain full decisional authority—with several variants inside each family (Table 1, Table 2.

**Table 1:** Summary of included studies.

**Study**	**Ref**	**Country**	**Uni/multicentric (n)**	**Study design**	**Baseline reading**	**Consensus / arbitration**	**Modality**	**Screens (n) †**
Dahlblom 2023	[15]	Sweden – Malmö	Unicentric	Retrospective simulation on prospective DBT trial	Double	Consensus	DBT (1-view)	14 772
Dembrower 2023 (ScreenTrustCAD)	[16]	Sweden – 4 county sites	Multicentric (4)	Prospective paired-reader non-inferiority	Double	None	DM	55 581
Elías-Cabot 2024	[17]	Spain – Córdoba	Unicentric	Pre-/post real-world cohort	Double	None	DM ± DBT	11 998
Heywang-Köbrunner 2023	[18]	Germany – Munich ref centre	Unicentric	Retrospective simulation, consecutive cohort	Double	Consensus	DM	17 884
Lång 2023 (MASAI)	[19]	Sweden – 4 screening units	Multicentric (4)	Randomised controlled non-inferiority	Double	Reader-initiated consensus permitted **‡**	DM	39 996
Lauritzen 2022	[20]	Denmark – Capital Region	Multicentric (4)	Retrospective consecutive-cohort simulation	Double	Arbitration	DM	114 421
Leibig 2022	[21]	Germany – 8 screening units	Multicentric (8)	Retrospective decision-referral simulation	Single §	None	DM	82 851
Letter 2023	[26]	USA – 3 academic sites	Multicentric (3)	Before–after service evaluation	Single	None	DBT	12 885
Marinovich 2023	[25]	Australia – BreastScreen WA	Multicentric (4)	Retrospective external validation + simulation	Double	Arbitration	DM	108 970
Seker 2024	[22]	Turkey – Bahçeşehir programme	Unicentric	Retrospective triage scenarios (10 y)	Double	Arbitration	DM	18 421
Sharma 2023	[23]	UK & HU – 7 sites	Multicentric (7)	Retrospective simulation of AI second reader	Double	Arbitration	DM	275 900
Elhakim 2023	[24]	Denmark – Southern Region	Multicentric (4)	Retrospective cohort simulation	Double	Arbitration	DM	257 671
Yala 2019	[27]	USA – single academic centre	Unicentric (1)	Retrospective deep-learning triage simulation	Single	None	DM	26 540

† “Screens (n)” corresponds to the number of examinations analysed in the AI arm. Some studies also included control groups; these were used for effect calculation but are not shown here to avoid double reporting.

‡ Women in the top 1 % of AI risk were recalled unless the radiologist identified an obvious false positive.

§ Only the first reader’s decision was used to model the decision-referral workflow; German programme normally uses double reading

**Table 2:** AI tool and integration in the screening workflow.

**Study (year)**	**AI product / version**	**Main AI use**	**Screening workflow**	**Detailed screening workflow**	**Threshold determination**	**Threshold details**
Dahlblom 2023	Transpara v1.7.0	Triage + Independent reader	T-GK-2R IND-ARB AI-only	3 different simulations: • AI triage: AI score ≤ 3 auto-negative; 3–9.99 double read • R1 +AI: AI score ≥ 8.74 forces consensus • Standalone AI: AI score ≥ 7.57 abnormal	Target-driven	score ≤ 3 chosen a-priori to auto-exclude ≈ 50 % of examinations, score 8.74 chosen a priori to generate the same number of consensus as real DBT double-reading
Dembrower 2023	Insight MMG v1.1.6	Independent reader	IND-ARB† AI-only	• R1 +AI: AI score ≥ 53.4 forces consensus • R1 +R2 +AI • Standalone AI	Externally preset	case-score ≥ 53.4 fixed on an external development set
Elías-Cabot 2024	Transpara v1.7.0	Concurrent CAD overlay	C-DS	• Concurrent decision‑support overlay visible to both radiologists in double-reading	No cut-off (display only)	Vendor default deciles (<30 low, 30–69 med, ≥70 high) for display only
Heywang-Köbrunner 2023	ProFound AI v2.0	Independent reader	IND-OR AI-only	• Standalone AI • AI + R1 / AI + R2 simulated (no consensus simulated‡)	Vendor default	case-score > 30 (ProFound AI v2) = positive
Lång 2023	Transpara v1.7.0	Triage	T-ALLOC	• AI triage: AI score 1–7: single reader without CAD marks, AI score 8–9: single reader with CAD marks visible, AI score 10: double-reading by two radiologists with CAD marks visible	Vendor default	Transpara risk decile 10 triggers double-reading; 1–9 single read
Lauritzen 2022	Transpara v1.7.0	Triage	T-DR-2R	• AI triage: AI score < 5: auto-neg, 5-recall threshold: double-read, > recall threshold: auto-recall.	Externally preset	recall-threshold 9.989 and skip < 5 fixed on dev cohort
Leibig 2022	Vara CNN	Triage	T-DR-1R	• AI triage: AI confident normal: auto-neg, AI confident abnormal: auto-recall, other: single reader	Internal-validation fixed	Low-risk cut at NT@0.97; high-risk cut at SN@0.98 tuned on internal validation
Letter 2023	ProFound AI v2.0	Concurrent CAD overlay	C-DS	• Concurrent decision‑support overlay visible to radiologist in single reading	No cut-off (display only)	ProFound marks and 0–100 score visible; no numeric rule
Marinovich 2023	Saige-Q v2.0	Independent reader	IND-ARB	• AI+R1 (simulated arbitration§)	Externally preset	Threshold fixed prospectively to give ≈ 4 % AI-positive rate
Seker 2024	Lunit INSIGHT MMG v1.1.7.1	Triage	IND-OR IND-ARB T-DR-1R	AI triage (AI results: green, orange or red) with 3 scenario: • Scenario 1: R1 +AI, no other reader (positive for R1 or red for AI) • Scenario 2: R1 +AI±R2, "flagged" exams (= positive for R1 or orange for AI) re-read by R2 • Scenario 3: AI±R1, AI auto-negate green exams, yellow exams are read by R1, AI auto-recall red exams	Post-hoc tuned	score ≥ 30.44 derived on test set by Youden index
Sharma 2023	Mia v2.0	Independent reader	IND-ARB	• AI+R1 (simulated arbitration§)	Vendor default	binary Mia v2 cut-off supplied by vendor, unchanged
El Hakim 2023	Transpara v1.7.0	Independent reader	IND-ARB	• AI+R1 (simulated arbitration§)	Externally preset	AIsens ≥ 9.56858 (match sens) & AIspec ≥ 9.71059 (match spec) fixed on external data
Yala 2019	ResNet-18 DL triage	Triage	T-GK-1R	• AI triage: Below cut-off: auto-neg; above cut-off: original single-reader decision.	Target-driven	cut-off = lowest AI score to covers all radiologists true positives on validation

Women in the top 1 % of AI risk were recalled unless the radiologist identified an obvious false positive.

† two modes: AI replacing the 2nd reader (double) and AI added to both radiologists (triple); in both cases discordant exams proceeded to the usual human consensus meeting

‡false-positive counts for the AI combinations represent the maximum possible recalls

§ AI replaces R2, discordances between R1 and AI leads to arbitration by historical arbitration or by R2 if missing

IND-OR: Independent AI reader + 1 radiologist; recall if AI OR R1 positive (no arbitration).

IND-ARB: Independent AI reader + 1 radiologist; AI–R1 discordance sent to second radiologist / consensus.

C-DS: Concurrent decision-support overlay; radiologist(s) view AI marks, AI casts no vote.

AI-only: Stand-alone AI, no human reader.

T-GK-1R: Gate-keeper triage: AI auto-negates “green” exams; remainder single-read by radiologist.

T-GK-2R: Gate-keeper triage: AI auto-negates “green” exams; remainder double-read by two radiologists.

T-DR-1R: Decision-referral triage: AI auto-neg + auto-recall; mid-risk exams single-read.

T-DR-2R: Decision-referral triage: AI auto-neg + auto-recall; mid-risk exams double-read.

T-ALLOC: Risk-allocation triage: AI never skips; it varies reader count by risk tier (e.g., low = 1 R, high = 2 R).

The commonest configuration was the independent-reader model with human arbitration (IND-ARB), evaluated prospectively or in simulation by Dembrower et al., Marinovich et.al, Sharma et al., Talal El Hakim et al., Dahlblom et al. (R1 + AI arm) and the “flagged” scenario in Seker et al. [15], [16], [22], [23], [24], [25]. In these studies the AI simply substituted one radiologist; a second breast-screening specialist still arbitrated discordant AI–human pairs. A leaner variant, IND-OR, removed that safety net and accepted an *either-positive* rule: if the AI or the single radiologist called the examination abnormal it was recalled. This approach was used in Heywang-Köbrunner et al. and Seker et al. scenario 1 [18], [22].

Two studies [17], [26] left decision-making entirely with the radiologist and supplied the AI only as a concurrent decision-support overlay (C-DS). Neither imposed a cut-off; heat-maps and continuous scores were shown on demand during the routine read.

Several groups explored gate-keeper triage in which very-low-risk examinations are auto-negated. When the remaining screens were single-read (T-GK-1R) the strategy was tested by Yala et al. [27]; when two radiologists still read the yellow and red tiers (T-GK-2R) it appeared in Dahlblom et al. [15]. The proportion of studies triaged away ranged from 20 % to 70 %.

In decision-referral triage (T-DR) the algorithm also issues an irrevocable auto-recall at the top end of its score range. A single-reader mid-tier (T-DR-1R) was examined by Leibig et al. and by Seker et al. scenario 3, whereas Lauritzen et al. kept double reading for the mid-risk band (T-DR-2R) [20], [21], [22].

MASAI [19] pioneered a risk-allocation (T-ALLOC) scheme: no exam was skipped. Score-10 cases underwent double reading with AI support, whereas score-1–9 cases received single reading with AI support (CAD marks displayed for scores 8–9).

Finally, four papers included a stand-alone AI (AI-only) benchmark [15], [16], [18], [21]. Although useful for technical comparison, these arms have no direct clinical analogue and were excluded from the pooled meta-analysis.

### 3.3 AI systems and operating thresholds

Nine different commercial algorithms were assessed. Three products—Transpara™ (ScreenPoint Medical), Mia™ (Kheiron Medical) and Lunit INSIGHT MMG—were used in seven of the twelve studies [16], [17], [19], [20], [22], [23], [24]. All studies applied either the vendor’s default operating point, or a pre-specified cut-off on an independent development set [19], [20], [25], except one study which used post-hoc threshold optimization [22].

### 3.4 Diagnostic accuracy versus conventional double reading

Table 3 shows individual study results of AI-augmented screening. The exact cancer and recall counts underlying the pooled analyses are listed in Table 4.

**Table 3:** Individual studies metrics.

**Study**	**Scenario / AI integration method**	**CDR AI / comparator (‰)**	**Statistical difference**	**Recall AI / comparator (%)**	**Statistical difference**
Dahlblom 2023	Gate-keeper triage (T-GK-2R)	8.19 / 8.60	P = 0.031	2.81 / 3.60	P < 0.001
Dahlblom 2023	Single reader + AI (IND-ARB)	8.12 / 8.60	P = 0.016	3.80 / 3.60	P = 0.054
Dembrower 2023	AI + R1 (IND‑ARB)	4.70 / 4.50	Non-inferior	2.80 / 2.93	0.45
Dembrower 2023	AI + R1 + R2 (triple)	4.84 / 4.50	p < 0.0001	3.1 / 2.93	0.03
Dembrower 2023	AI-only	4.43 / 4.50	Non-inferior	1.55 / 2.93	P < 0.05
Elias-Cabot 2024	Concurrent overlay (C-DS)	9.00 / 5.83	—	6.10 / 5.40	—†
Heywang-Köbrunner 2023	AI OR R1 (IND-OR)	6.43 / 6.43	—	25.45 / 13.36 ‡	—
Lång 2023	Risk allocation (T-ALLOC)	6.10 / 5.07	—	2.15 / 2.04	—
Lauritzen 2022	Decision-referral (T-DR-2R)	6.81 / 6.91	—	2.06 / 2.53	—
Leibig 2022	Decision-referral (T-DR-1R)	30.27 / 29.44	—	8.53 / 9.32	—
Letter 2023	Before–after overlay (C-DS)	7.31 / 5.86	P = 0.16	11.69 / 11.80	P = 0.92
Marinovich 2023	Prospective threshold 1 (IND-ARB)	6.37 / 6.97	—	3.14 / 3.38	—
Seker 2024	Scenario 1 – IND-OR	4.56 / 3.69	P = 0.12	6.12 / 6.34	P = 0.45
Seker 2024	Scenario 2 – IND-ARB	5.48 / 3.69	P = 0.02	5.91 / 6.34	P = 0.30
Seker 2024	Scenario 3 – T-DR-1R	5.43 / 3.69	P = 0.03	4.48 / 6.34	P = 0.001
Sharma 2023	Multi-vendor (IND-ARB)	9.72 / 10.12	—	2.80 / 2.80	—
Elhakim 2023	Aisens (IND‑ARB)	5.53 / 5.74	P = 0.58	3.36 / 2.70	P = 0.01
Elhakim 2023	AIspec (IND‑ARB)	5.13 / 5.74	P = 0.10	2.71 / 2.70	P = 0.45
Yala 2019	Gate-keeper triage (T-GK-1R)	6.48 / 6.52	—	6.43 / 7.15	—

† For Elías-Cabot et al., p-values are reported in the original publication for global and modality-specific matched analyses (DM and DBT), but not for each individual metric in the format shown here; therefore, no p-value is displayed.

‡ The unusually high recall rates in Heywang-Köbrunner 2023 reflect the characteristics of the local screening population, which included a high proportion of subtle cancers, dense breasts, and mandatory recalls (e.g., for palpable findings), as detailed by the authors.

CDR: cancer detection rate

**Table 4:** Characteristics of AI-Augmented Screening Arms Included in the Meta-Analysis.

**Study (year)**	**Scenario / AI integration method**	**AI role**	**Screens (AI / comp)**	**Screen-detected cancers (AI / comp)**	**Recalls (AI / comp)**	**Note**
Dahlblom 2023	Gate‑keeper triage (T‑GK‑2 R)	Triage	14 772 / 14 772	121 / 127	415 / 532	a
Dembrower 2023	R1 + AI (IND‑ARB)	Independent	55 581 / 55 581	261 / 250	1 556 / 1 629	b
Elías‑Cabot 2024	Real‑world overlay (C‑DS)	Concurrent	11 998 / 11 998	108 / 70	732 / 648	c
Heywang‑Köbrunner 2023	AI OR R1 (IND‑OR)	Independent	17 884 / 17 884	115 / 115	4 551 / 2 390	a
Lång 2023 (MASAI)	Risk‑allocation (T‑ALLOC)	Triage	39 996 / 40 024	244 / 203	861 / 817	
Lauritzen 2022	Decision‑referral, double mid‑tier (T‑DR‑2 R)	Triage	114 421 / 114 421	779 / 791	2 357 / 2 898	
Leibig 2022	Decision‑referral, single reader (T‑DR‑1 R)	Triage	82 851 / 82 851	2 508 / 2 439	7 071 / 7 723	d
Letter 2023	Before–after overlay (C‑DS)	Concurrent	5 883 / 7 002	43 / 41	688 / 826	c
Marinovich 2023	Prospective threshold 1 (IND‑ARB)	Independent	108 970 / 108 970	694 / 760	3 417 / 3 684	e
Seker 2024	Scenario 2 – IND‑ARB	Independent	18 421 / 18 421	101 / 68	1 089 / 1 168	f
Sharma 2023	AI as 2nd reader (IND‑ARB)	Independent	275 900 / 275 900	2 683 / 2 792	7 725 / 7 725	g
Elhakim 2023	Integrated AIₛₑₙₛ (IND‑ARB)	Independent	257 671 / 257 671	1 425 / 1 479	8 664 / 6 956	
Yala 2019	Gate‑keeper triage (T‑GK‑1 R)	Triage	26 540 / 26 540	172 / 173	1 707 / 1 897	d

a. Other arms not included in the meta-analysis: stand-alone and R1 +AI.

b. Triple-reading arm omitted to avoid double-counting the same screens.

c. Recall counts back-calculated from published recall rates × denominator.

d. Recall counts reconstructed from reported sensitivity/specificity and cohort size.

e. Higher-specificity threshold arm excluded; authors designate threshold 1 for practice.

f. Recall counts derived from 'flagged-visit' totals; denominator 18 421 screens.

g. Counts pooled across seven vendors (Supplement S2); vendor-specific arms not analysed separately.

**Fig. 2:**
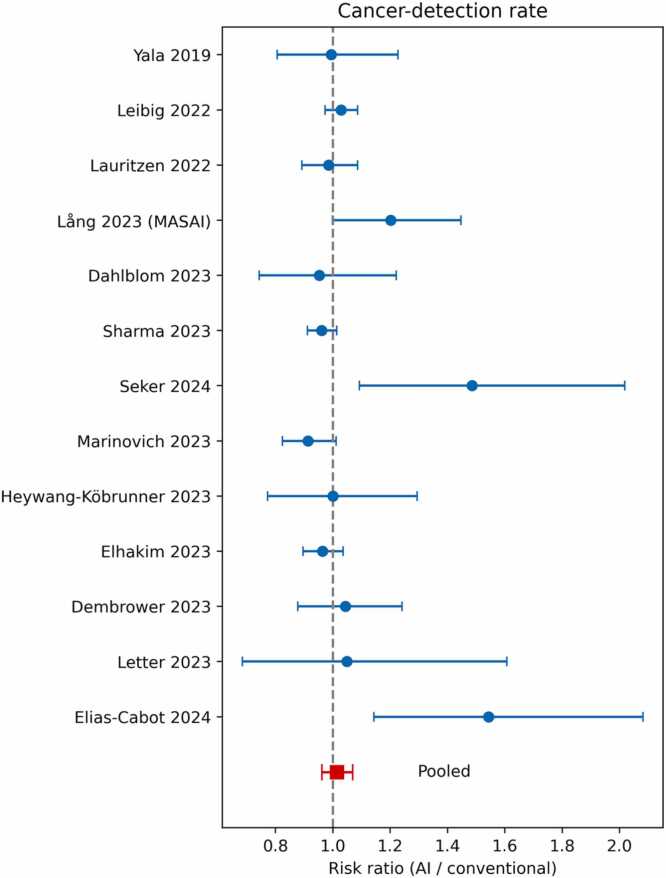
Forest plot of the relative cancer-detection rate for artificial intelligence (AI)-augmented reading versus conventional double-reading. Each blue dot represents the risk ratio (RR) from an individual study, with the horizontal lines indicating the 95 % confidence interval. The red square represents the pooled RR for all studies combined. The dashed vertical line at RR = 1.0 indicates no difference between the two reading methods.

**Fig. 3:**
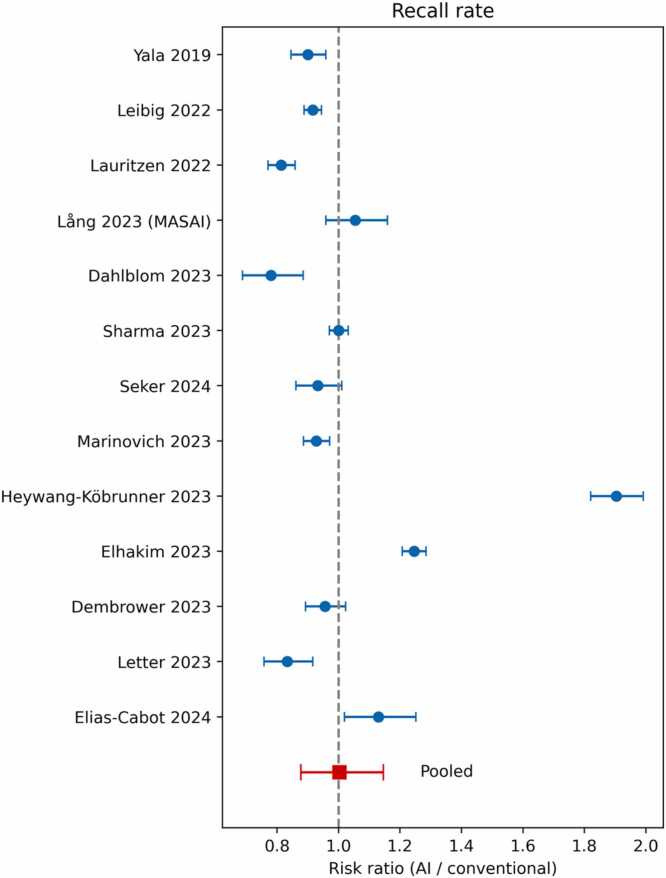
Forest plot of the relative recall rate for artificial intelligence (AI)-augmented reading versus conventional double-reading. Each blue dot represents the risk ratio (RR) from an individual study, with the horizontal lines indicating the 95 % confidence interval. The red square represents the pooled RR for all studies combined. The dashed vertical line at RR = 1.0 indicates no difference in recall rates between the two methods.

Across 13 non-overlapping study arms (≈ 1.03 million screening examinations) AI did **not** significantly change programme sensitivity. The pooled risk ratio (RR) for screen-detected cancers was 1.01 (95 % CI (Confidence Interval) 0.96–1.07; heterogeneity index I² = 53 %), indicating virtual parity with conventional double reading (Fig. 2). Recalls likewise remained unchanged overall—RR 1.00 (0.88–1.15)— (Fig. 3) but heterogeneity was extreme (I² ≈ 99 %), signalling that the direction of recall change might depend on how the algorithm is inserted into the workflow.

Studies were therefore grouped by integration model, such as mentioned in Table 4. The corresponding arm-selection process for studies with multiple scenarios is presented in Supplementary Figure S1.

Triage workflows (gate-keeper [15], [27], decision-referral [20], [21] and MASAI-type risk allocation [19]) preserved cancer detection—RR 1.02 (0.98–1.07)—while reducing recalls by 11 %—RR 0.89 (0.82–0.96). Between-study heterogeneity in CDR disappeared (I² = 0 %), indeed each study calibrated its triage threshold to preserve the programme’s historical sensitivity, so point estimates clustered tightly around unity; oppositely, variability in recall (I² ≈ 87 %) reflected how aggressively each study skipped low-risk exams.

Independent AI readers (algorithm replaces one human vote) [16], [18], [22], [23], [24], [25] left CDR statistically unchanged—RR 0.98 (0.92–1.05)—but recall effects ranged from a modest fall to a 90 % rise, yielding a non-significant average increase—RR 1.12 (0.90–1.39) and I² > 99 %. The outliers were studies using an “either-positive” rule, underscoring that recall burden in this configuration is driven by the chosen arbitration logic and AI threshold.

Concurrent overlays (AI marks shown during a single read, both in DBT settings) hinted at a sizeable sensitivity gain—RR 1.31 (0.90–1.91)—with no recall penalty—RR 0.97 (0.72–1.31), but only in two U.S. studies [17], [26], and with high heterogeneity.

#### 3.4.1 Workload reduction

Results across the 19 AI-integration arms are summarised in Table 5. Two consistent patterns emerge.

**Table 5:** Workload change reported by each study.

**Study**	**Scenario / AI integration method**	**Δ initial reads**	Δ arbitration*
Dahlblom 2023	Gate‑keeper triage (T‑GK‑2 R)	−49 %	−28 %
Dahlblom 2023	Independent 2nd reader (IND‑ARB)	−50 %	≈ 0 % (threshold-matched)
Dahlblom 2023	AI stand‑alone	−100 %	—
Dembrower 2023	AI + R1 (IND‑ARB)	−50 %	−21 %
Dembrower 2023	AI + R1 + R2 (triple)	0 %	+ 38 %
Elías‑Cabot 2024	Concurrent overlay (C‑DS)	0 %	0 %
Heywang‑Köbrunner 2023	R1 + AI (IND‑OR)	−50 %	+ 100 %
Heywang‑Köbrunner 2023	AI‑only (research)	−100 %	—
Lång 2023 (MASAI)	Risk‑allocation triage (T‑ALLOC)	−44 %	≈ 0 %
Lauritzen 2022	Decision‑referral (T‑DR‑2 R)	−63 %‡	—
Leibig 2022	Decision‑referral (T‑DR‑1 R)	−63 %‡	—
Letter 2023	Concurrent overlay (DBT)	0 %	—
Marinovich 2023	Threshold 1 (IND‑ARB)	−50 %	+ 305 % (+15 pp)
Marinovich 2023	Threshold 2: higher specificity (IND‑ARB)	−50 %	+ 113 % (+5pp)
Seker 2024	Scenario 1 IND‑OR	−50 %	—
Seker 2024	Scenario 2 IND‑ARB	−23 %	+ 32 %
Seker 2024	Scenario 3 T‑DR‑1 R	−70 %	−29 %
Sharma 2023	Multi‑vendor (IND‑ARB)	−50 % 2nd reads	+ 270 %
El Hakim 2023	AIsens cut‑off (IND‑ARB)	−50 %	+ 78 % (+2.2pp)
El Hakim 2023	AIspec cut‑off (IND‑ARB)	−50 %	+ 38 % (+1.1pp)
Yala 2019	Gate‑keeper triage (T‑GK‑1 R)	−19 %‡	—

*Δ arbitration = percentage change in number of exams sent to consensus/arbitration relative to the study’s baseline double-reading workflow.

‡Value is percentage of screens auto-dismissed; arbitration not reported.

“—” indicates the study did not simulate or publish arbitration data.

Gate-keeper or decision-referral triage cut the number of initial reads by 44 – 70 % and either reduced or left unchanged the arbitration burden [15], [19], [20], [21], [22], [27].

Conversely, AI replacing one of the independent readers halved first reads but generated a pronounced surge in arbitration, with increases of + 270 % [23] to more than + 300 % [25], depending on calibration strategy. Marinovich et al. [28] also observed a doubling of arbitrations in their simulated AI–radiologist workflow. In Dahlblom et al., no independent-reader-arbitration pathway was evaluated, and arbitration effects were therefore not estimable [15]. Threshold optimisation [24], [25] attenuated—but did not eliminate—the arbitration surge. Finally, concurrent overlays leave staffing unchanged [17], [26].

### 3.5 Risk-of-bias assessment

**Fig. 4:**
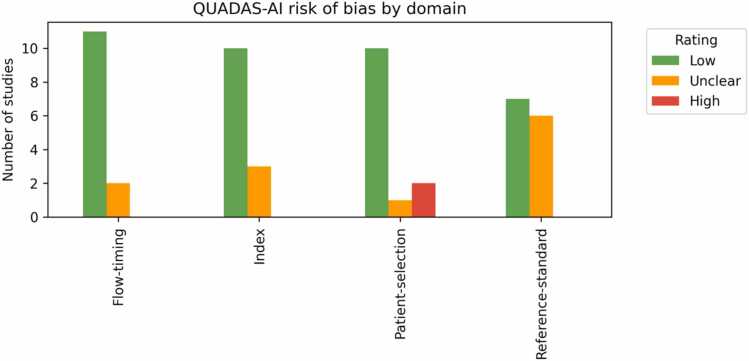
Summary of the risk-of-bias assessment for the 13 included studies using the QUADAS-AI tool. The bars represent the number of studies judged to have low (green), unclear (orange), or high (red) risk of bias across four key domains. QUADAS-AI stands for Quality Assessment of Diagnostic Accuracy Studies–Artificial Intelligence.

A graphical summary of QUADAS-AI assessment is provided in Fig. 4. Across the 13 primary studies (19 AI arms), most domains were judged low risk, with concerns clustering in only two areas.

*Flow & timing.* Eleven studies (85 %) linked every screen to its final diagnostic outcome and included interval cancers; in two studies, the pre-post design or the absence of simulation of the consensus yielded an unclear rating.

*Index-test conduct*. Three simulation studies did not lock the AI threshold prospectively, so their Index domain was scored unclear; all other trials pre-specified the operating point and were thus considered low risk.

*Patient selection.*Two enriched or convenience cohorts [21], [23] were flagged high risk because they excluded a sizeable share of routine screens. One additional study had insufficient detail on inclusions and exclusions [27]; the other ten were consecutive population cohorts.

*Reference standard*. Verification bias is the main limitation: only seven studies (54 %) linked screen negatives to a regional or national cancer registry; six reported screen-detected cancers only, giving an unclear rating.

## 4 Discussion

Across more than one million screening examinations, our meta-analysis confirms that AI can be safely embedded into organised breast-screening without compromising programme sensitivity: the pooled cancer-detection rate (CDR) was essentially identical to conventional double reading (RR 1.01, 95 % CI 0.96–1.07). However, recall outcomes varied markedly by integration model. Gate-keeper and decision-referral triage workflows reduced recalls by an average of 11 % while excising low-risk examinations and cutting initial radiologist reads by 44–70 %. By contrast, independent-reader configurations halved routine reads but, depending on arbitration logic and AI–human discordance, drove recall changes ranging from –20 % to + 90 %.

Within each integration-model family, screen-detected cancer counts also varied in both directions (Table 4). In triage studies (Δ –0.41 to +1.03 per 1000; RR 0.95–1.20), differences were small and largely aligned with each study’s threshold calibration: Dahlblom and Yala remained effectively neutral, Lauritzen showed minimal deviation, Leibig showed a modest gain consistent with DBT availability, and the MASAI study was the only clear outlier (RR 1.20) due to explicit sensitivity-preserving risk allocation. Independent-reader designs showed wider dispersion (Δ –0.61 to +1.79 per 1000; RR 0.91–1.49), reflecting genuine methodological heterogeneity: arbitration logic (consensus vs. “either-positive”), threshold selection, DBT–DM mix, and differences in case ascertainment (registry linkage, interval-cancer inclusion, reconstructed ground truth). Concurrent overlays (Δ +1.45 to +3.17 per 1000; RR 1.25–1.54) consistently increased detection but are represented by only two heterogeneous studies. Overall, these bidirectional variations in Table 4 primarily reflect differences in case ascertainment and workflow design—not contradictory effects of AI—and explain why triage models converge to RR ≈ 1.0 (I² = 0 %) whereas independent-reader models show broader dispersion.

Our findings build on the stand-alone meta-analysis by Yoon et al. [4] by showing that matching radiologist AUC in isolation does not guarantee workflow efficiencies once AI is integrated into a double-reading pathway. Indeed, the MASAI (Mammography Screening with Artificial Intelligence) trial [19] – confirmed by a second analysis [29] – demonstrated that a risk-allocation triage protocol halved reader workload without any recall penalty, while significant increasing cancer detection (mainly small, lymph-node negative breast cancer). In the ScreenTrustCAD trial [16] replacing the second reader with AI achieved non-inferior cancer detection (relative proportion 1.04, 95 % CI 1.00–1.09) and halved the number of independent reads—but when a third human reader was retained for arbitration, the consensus caseload rose by 38 %.

In settings where same-day supplementary ultrasound is routine, real-world single-reader assistance with AI-CAD did not significantly change sensitivity, AUC or recall. [30]

Two recent large-scale simulations confirm these implementation-dependant differences. A Danish simulation study [31] compared three AI-integration strategies and found that only the triage approach consistently maintained or improved cancer detection and reduced both reading and arbitration workloads. In an extension of Leibig’s decision-referral concept across nearly 1.9 million UK, German and Swedish exams, programme-level decision-referral triage delivered the greatest detection gain (+8.3 % CDR) and work-load reduction (–84 %) compared to standalone or simple gate-keeper models [32].

Several general conclusions emerge. First, every triage study that auto-dismissed low-risk exams saved at least 40 % of radiologist reads without triggering an arbitration surge; such models suit programmes under severe workforce pressure, provided robust safety nets (interval-cancer audits, sensitivity minimums) are in place. Second, independent-reader models currently lack prospective validation. An AI threshold that minimises AI-human discordance is essential; otherwise, the arbitration phase becomes the new bottleneck. In a recently published study, Marinovich et al. showed retrospective arbitration underestimates both CDR and recall impacts [28], reinforcing our recommendation that prospective, live arbitration data—not reconstructed simulations—be used to calibrate AI thresholds and monitor discordance in deployment. Third, concurrent CAD overlays do not, in the configurations evaluated here, reduce the number of cases each radiologist must read; however, they can accelerate per-case interpretation. In the recently published PRAIM nationwide study, median read time fell by 43 % (from 67 s to 39 s), alongside a 17.6 % CDR increase and unchanged recall [33]. Although a “single reading with overlay” configuration could theoretically replace double reading, this mode has not yet been prospectively evaluated outside of triage-based frameworks such as the intermediate-risk arm of MASAI.

Our review’s strengths include its exclusive focus on population-based cohorts or pragmatic trials (avoiding enriched datasets), clear stratification by integration model, and extraction of absolute cancer and recall counts for uniform effect measures. Limitations mirror our risk-of-bias assessment: six studies lacked interval-cancer follow-up, two used convenience samples, and nearly half were retrospective simulations rather than live deployments. Although recall and arbitration heterogeneity was high—reflecting varied thresholds, reader training and governance—the direction of effect within each integration family proved consistently reliable.

Key gaps remain. First, prospective, multi-centre evaluations of AI-driven triage remain rare, and real-world pilots will be essential to confirm whether the substantial workload reductions seen in simulations are persistent with time. Equally important is the routine inclusion of interval-cancer capture in study endpoints to guard against any subtle sensitivity losses. We also lack detailed insights into reader–AI interactions—understanding why discordance rates vary by vendor, threshold and reader experience will be critical to refining deployment strategies. Comprehensive health-economic analyses that link radiologist time savings and recall consequences to downstream costs and patient outcomes are urgently needed to inform reimbursement and implementation policies. Finally, equity and acceptability must remain foremost: qualitative work shows the public supports AI so long as human oversight and accuracy are preserved [5], [6], [7], and UK breast-screening readers favour partial replacement of one reader by AI while rejecting full automation, preferring graphical region-of-suspicion displays, all within a guideline-driven rollout framework [34].

## 5 Conclusion

AI can safely support population breast-cancer screening but not all deployments are equal. Triage protocols consistently halve radiologist workload without compromising detection, whereas swapping an algorithm for the second human may simply displace labour to the arbitration room. Regulatory guidance and service roll-outs should therefore pivot from headline accuracy to workflow-aware performance metrics—sensitivity, recall, and net human reads—reported side-by-side.

## CRediT authorship contribution statement

**Catherine Roy:** Supervision, Project administration, Conceptualization. **SOSSAVI Eloïse:** Writing – original draft, Methodology, Investigation, Formal analysis, Conceptualization. **Sébastien Molière:** Validation, Project administration, Methodology, Formal analysis, Conceptualization.

## Ethics

All procedures were performed in compliance with relevant laws and institutional guidelines and have been approved by the appropriate institutional committee

## Funding

This research did not receive any specific grant from funding agencies in the public, commercial, or not-for-profit sectors.

## Declaration of Competing Interest

The authors declare that they have no known competing financial interests or personal relationships that could have appeared to influence the work reported in this paper.
