# Supplementary material for: Artificial intelligence in breast cancer screening: A systematic review and meta-analysis of integration strategies
Source: Eur J Radiol Open. 2026 Jan 10;16:100727. doi: 10.1016/j.ejro.2026.100727 (PMC12818982; doi:10.1016/j.ejro.2026.100727)
Supplement: Supplementary file 1 — Supplementary material [file mmc1.docx]

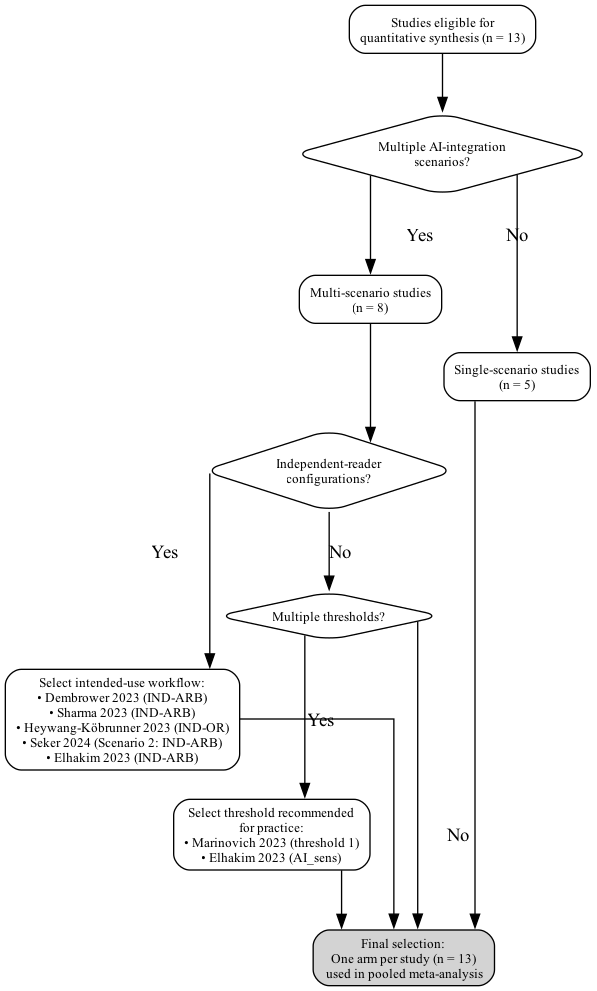


Supplementary Figure S1. Selection process for choosing a single AI-integration arm per study for the meta-analysis. When studies reported several scenarios based on the same underlying screening cohort, we retained only the configuration designated by the authors as their intended-use workflow. In threshold-based designs, we selected the threshold recommended for clinical practice. Single-scenario studies were included as-is. This approach prevents double-counting and ensures comparable clinical intent across pooled analyses.
